# Etiology and clinical features of Han Chinese patients with Duane retraction syndrome

**DOI:** 10.3389/fgene.2025.1500090

**Published:** 2025-03-27

**Authors:** Lijuan Huang, Baoying Chen, Chi Cai, Yuyu Wu, Zhimin Sun, Yan Xie, Ningdong Li

**Affiliations:** ^1^ Department of Ophthalmology, The Second Affiliated Hospital of Fujian Medical University, Quanzhou, China; ^2^ Department of CT/MRI, The Second Affiliated Hospital of Fujian Medical University, Quanzhou, China; ^3^ Department of Ophthalmology, Second Affiliated Hospital of Nantong University, Nantong, China; ^4^ Department of Ophthalmology, Beijing Children’s Hospital, Capital Medical University, Beijing, China; ^5^ Department of Ophthalmology, Shanghai General Hospital, Shanghai, China

**Keywords:** Duane retraction syndrome, etiology, treatment, *CHN1*, *SALL4*

## Abstract

Duane retraction syndrome (DRS) is a congenital ocular motility disorder. The aim of this study was to retrospectively describe the etiology, clinical findings, imaging characteristics, and surgical outcomes of 42 Han Chinese patients with DRS. All patients underwent detailed clinical evaluation. Next-generation sequencing was performed to identify pathogenic variants in the disease-causing genes. Magnetic resonance imaging (MRI) and diffusion tensor imaging (DTI) tractography were used to evaluate the patient’s cranial nerves. Surgical procedures were designed individually to correct strabismus, abnormal facial turns, and overshooting. A total of 17 patients were diagnosed with DRS1, 4 with DRS2 and 21 with DRS3. Genetic testing revealed that two novel pathogenic variants of c.377T>C (p. Ile126Thr) and c.659A>G (p. Glu220Gly) in the *CHN1* gene and a *de novo* pathogenic variant of c.1432-2A>T in the *SALL4* gene were detected in patients with DRS1. In 12 of the 14 patients with DRS1 and 9 of the 17 patients with DRS3, the abducens nerve was found to be absent in the MRI images, and in 4 of the patients with DRS2, the abducens nerve was detected as hypoplasia. In addition, the projective fibers from the abducens neurons to the contralateral ocular motor neurons via the medial longitudinal fasciculus were also absent in those patients without abducens nerve in DTI images. Thirty-five patients who underwent strabismus surgery gained binocular vision and an improved appearance. In summary, our genetic findings contribute to expanding the spectrum of variants in the *CHN1* and *SALL4* genes. Molecular etiology and imaging studies support that cranial maldevelopment is a major cause of DRS. Individualized treatment based on ocular movement can effectively improve the symptoms and signs of patients with DRS.

## 1 Introduction

Duane retraction syndrome (DRS) refers to a group of ocular motility disorders characterized by absolute restriction on abduction and relative restriction on adduction, with retraction of the eyeball and narrowing of the palpebral fissure on adduction (Muni and Kumar, 2023; Ahluwalia et al., 1988). Based on electromyography and clinical features, DRS may be classified into forms of DRS1, DRS2, and DRS3 (Huber, 1974).

DRS1 is the most common form, followed by DRS3 and DRS2 (Kekunnaya and Negalur, 2017). It is characterized by complete restriction on abduction and esotropia at the primary gaze position. Patients with DRS1 often present with a compensatory face turn in the direction of the affected eye in order to obtain binocular vision. DRS2 is characterized by a severe limitation on adduction and a large exotropia. Patients with DRS2 may have a compensatory face turn away from the direction of the affected eye. DRS3 is characterized by restriction on both ab- and ad-ductions and may present as orthotropic, slight eso- or exo-tropia depending on the equilibrium between the tight medial and lateral rectus muscles. Whether or not having a compensatory head position in patients with DRS3 depends on their ocular alignment at the primary eye position (Mohan et al., 2008). Patients with DRS2 and DRS3 may have their eyeball upshoot and downshoot which are usually caused by simultaneous contraction of both tight medial and lateral muscles on adduction, denoted as the “bridle effect” (Von Noorden and Murray, 1986). DRS may occur in one or both eyes, with unilateral involvement being more common than bilateral involvement. Moreover, an extremely rare type of DRS may occur, named DRS4, and is characterized by a synergistic divergence of both eyes due to the affected eye being paradoxically abducted when attempted adduction, which is caused by misrouting of the ocular motor never to the lateral rectus muscle (Schliesser et al., 2016; Shen et al., 2021).

The etiology of DRS is related to the dysgenesis of the sixth cranial nucleus or nerve, so it belongs to congenital cranial dysinnervation disorders (CCDDS). Some patients with DRS have a family history showing as an autosomal dominant (AD) trait, however, a majority of patients are sporadic. To date, three genes have been identified to be associated with DRS, including *chimerin 1* (*CHN1,* OMIM*118423) on chromosome 2q31 (Miyake et al., 2008), *spalt-like transcription factor 4* (*SALL4,* OMIM*607323) on chromosome 20q13.13-13.2 (Al-baradie et al., 2002), *MAF bZIP transcription factor B* (*MAFB,* OMIM*608968) on chromosome 20q12 (Park et al., 2016). These genes have been shown to be involved in neuronal development.

Surgery is an effective means in correcting the ocular misalignment in the primary eye position, improving a compensatory head posture, increasing abduction, enlarging the binocular visual field, and reducing the globe shoots (Kekunnaya et al., 2015; Akbari and Mirmohammadsadeghi, 2017). Surgical methods include recession of the tight medial rectus (MR) or lateral rectus (LR) muscle, vertical rectus muscle transposition (VRT) for an increase of abduction, LR recession in conjunction with LR Y-splitting, or simultaneous recession of MR and LR for treatment of shoots. Here, we retrospectively review clinical features, including pre- and post-operative features, molecular etiologies, and image features in a cohort of patients with DRS.

## 2 Materials and methods

### 2.1 Patients

Medical records were reviewed retrospectively for 42 patients with DRS between March 2021 and March 2024. DRS is diagnosed based on the following signs in one or both eyes: limitation on ab- and ad-duction, narrowing of the palpebral fissure on adduction and retraction of the affected eyeball. Patients with DRS underwent general ocular examinations, including their best corrected visual acuity, anterior segment and fundus. Ocular alignment and motility were evaluated carefully. Thirty-eight patients are sporadic and 4 patients have a family history of DRS. Thirty-one patients were diagnosed with unilateral DRS, with 60% involvement in the left eye and 40% in the right eye. Eleven patients had bilateral DRS. Seventeen patients were classified as DRS1, four as DRS2 and 21 as DRS3. In addition to limited horizontal mobility, a few patients have nystagmus (n = 4), crocodile tears (n = 1), radial bone malformations (n = 1), optic nerve hypoplasia (n = 1), caries (n = 1), and dysgenesis of the brainstem (n = 1).

### 2.2 Etiology analysis

Etiology analysis included the genetic testing and imaging examinations by high-resolution magnetic resonance imaging (MRI) using a 3.0 T system (Achieva, Philips Medical Systems, Best, Netherlands). The MRI images were acquired using a three-dimensional (3D) T1-weighted magnetization prepared rapid gradient echo sequence covering the brain with the following parameters: repetition time (TR)/echo time (TE) = 8.3 ms/3.8 ms; flip angle = 12^◦^; field of view = 180 × 180 mm^2^; acquisition matrix = 180 × 180; slice = 164; slice thickness = 1 mm; and voxel size = 0.5 × 0.5 mm^2^. Axial diffusion tensor imaging (DTI) was performed using an echo-planar imaging sequence: TR/TE = 9,300 ms/100 ms; 30 diffusion-weighted directions with a b-value of 1,000 s/mm^2^; and a single image with a b-value of 0 s/mm^2^, slice thickness of 2 mm, slice gap of 0 mm, 68 slices, acquisition matrix of 128 × 128, and an FOV of 256 × 256 mm^2^. DTI tractography was performed using a Philips IntelliSpace portal.

Molecular etiology was analyzed through genetic testing using the panel-based next-generation sequencing commercially performed by the Mygenomic Biochemical Company (Beijing, China). Peripheral blood samples were taken from all participants with their informed consent. Genomic DNA was extracted, and a DNA library was constructed. Sequencing was performed on an Illumina HiSeq X Ten platform (Illumina, San Diego, CA). Raw data was collected and mapped to the human reference genome GRCh38. Variants were analyzed using a Genome Analysis Toolkit (GATK), and searched in at least four databases including dbSNP151, EXAC, gnomAD 2.1, ClinVar and HGMD2021. The candidate variants were validated using Sanger bidirectional sequencing. Pathogenicity of the candidate variants were evaluated using the online programs of SIFT, PolyPhen-2, Combined Annotation Dependent Depletion (CADD), Mutation Taster, and the guidelines enacted by the American College of Medical Genetics and Genomics (ACMG) and the Association for Molecular Pathology (AMP) (ACMG/AMP). The splicing effect of the variants was evaluated using the SpliceAI program. Mutations were named following the nomenclature recommended by the Human Genomic Variation Society (HGVS). Protein 3D structures were modelled using the AlphaFold Protein Structure Database (https://alphafold.ebi.ac.uk) and visualized using the PyMol program (https://pymol.org). The DynaMut (https://biosig.lab.uq.edu.au/dynamut/) was used to predict the protein stability. This study was conducted in accordance with the tenets of the Helsinki Declaration.

### 2.3 Surgery

MR recession was performed to correct esotropia in conjunction with superior rectus muscle transposition (SRT) to amplify abduction for the patients with DRS1 (Doyle and Hunter, 2019). An augmented LR recession was performed to correct a large angle exodeviation in DRS2. The globe retraction and shoots were corrected using two different surgical procedures, including Y-splitting of LR, simultaneous recession of MR and LR (Arcot Sadagopan et al., 2023). All surgeries were performed by a senior surgeon (Dr Li). Surgical success was defined as a horizontal deviation of <10^△^ in primary position at the follow-up of 3 months.

### 2.4 Statistical analysis

The pre- and postoperative alignment were compared with nonparametric tests using SPSS version 24.0 (SPSS, Chicago, United States). A *P* value of <0.05 was considered statistically significant.

## 3 Results

### 3.1 Genetic findings

**FIGURE 1:**
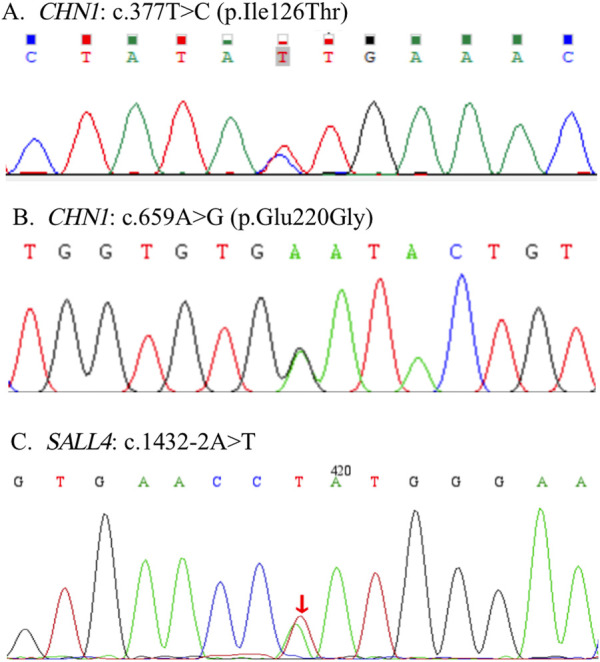
Three novel pathogenic variants were detected in this study. Two pathogenic variants of c.377T>C (p. Ile126Thr) and c.659A>G (p. Glu220Gly) were detected in the *CHN1* gene **(A, B)**. A *de novo* pathogenic variant of c.1432-2A>T was detected in the *SALL4* gene **(C)**.

**FIGURE 2:**
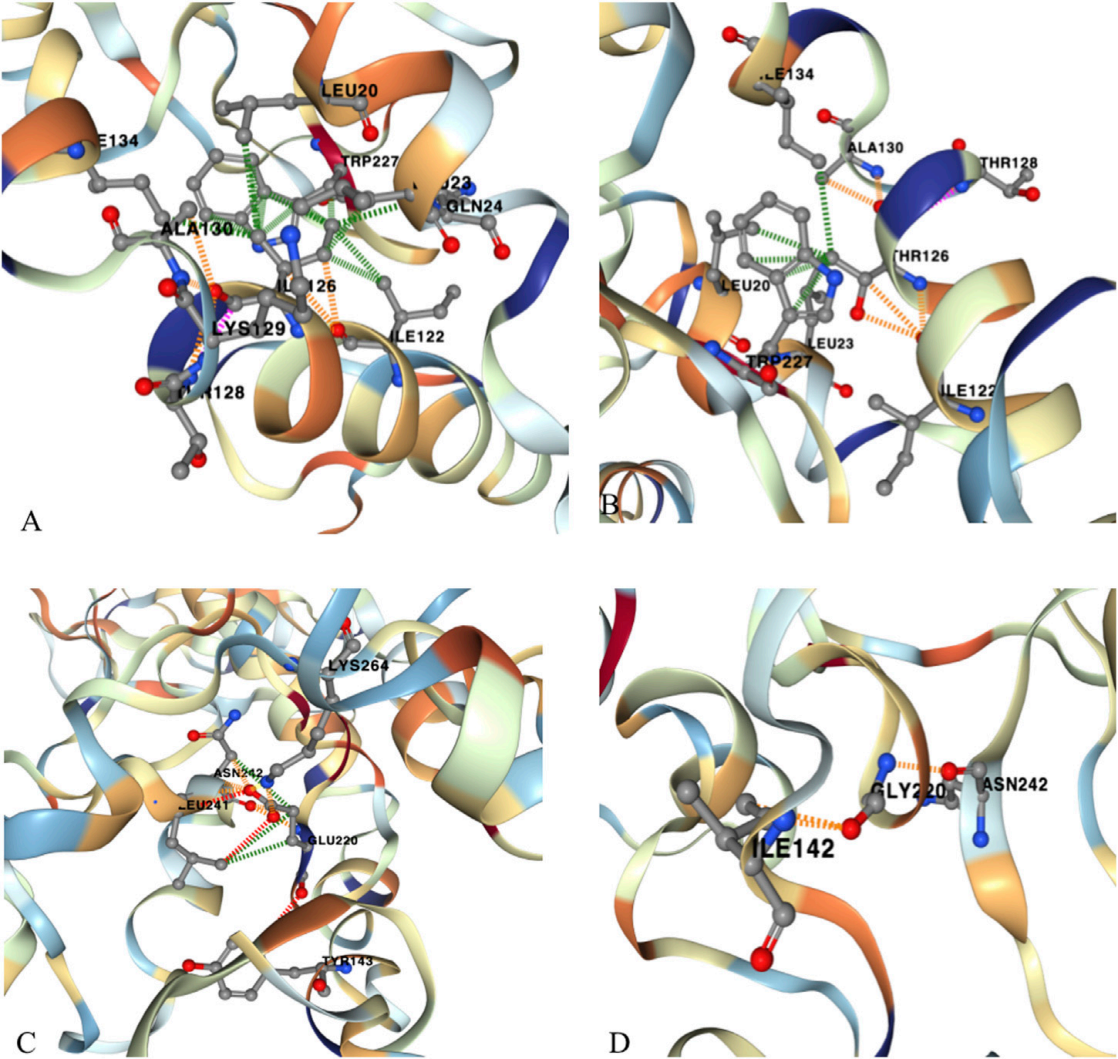
Three-dimensional model construction for novel missense variations of c.377 T>C (p. Ile126Thr) and c.659A>G (p. Glu220Gly) in *CHN1*. The model shows that a wild-type non-polar amino acid of Isoleucine (Ile) was replaced by a polar amino acid of Threonine (Thr) at codon 126, which would make the connective hydrogen bands get lost between Isoleucine and Lysine (Lys) at codon 129. **(A)** wild type; **(B)** mutant type. Wild-type Glutamic Acid (Glu) was replaced by Glycine (Gly) at codon 220, which would make the connective hydrogen band get lost to TYR143 and to Leu241, and to Lys264, and make abnormal connections to Ile142. **(C)** wild type; **(D)** mutant type. These changes may impair the stability of the protein structure and function.

The variants of c.377T>C (p. Ile126Thr) and c.659A>G (p. Glu220Gly) in *CHN1* were detected from two pedigrees of NT1 and CF1with DRS1. The NT1 pedigree included the affected girl who is the proband and her affected mother. Both carried with c.377T>C (p. Ile126Thr) variant (Figure 1A). The CF1 pedigree also included two affected members, where the affected mother and her daughter both carried the c.659A>G (p. Glu220Gly) variant (Figure 1B). In addition, a *de novo* splicing variant of c.1432–2A>T in the *SALL4* gene was found from a sporadic patient with DRS1 (Figure 1C). The missense variation of c.377T>C (p. Ile126Thr) and c.659A>G (p. Glu220Gly) were predicted to be deleterious to the function and structure of the protein by *in silico* analysis using SIFT, Polyphen-2, Mutation Taster, and CADD (Table 1), and were further confirmed by three-dimensional model construction using the PyMOL program (Figure 2). The SpliceAI program predicted a delta score of zero for the splicing variant of c.1432-2A>T.

**TABLE 1:** Novel variations in patients with Duane retraction syndrome in this study.

Gene	Location	Nucleotide	Protein	Type	SIFT	Mutation Taster	CADD	ACMG	Evidence levels	SpliceAI
*CHN1*	Exon6	c.377T>C	p. Ile126Thr	Missense	D	D	D	VUS	PM2 + PP4	—
*CHN1*	Exon8	C.659A>G	p. Glu220Gly	Missense	D	D	D	VUS	PVS1 + PM2 + PP4	—
*SALL4*		c.1432-2A>T		Splicing	—	—	—	Pathogenic	PVS + PS2 + PM2	0

Nucleotide annotation and exons numbering were based on reference sequences NM_001822 (*CHN1*) and NM_020436 (*SALL4*). Abbreviation: D - damaging/Disease_causing. VUS: variants of uncertain significance.

### 3.2 MRI findings

**FIGURE 3:**
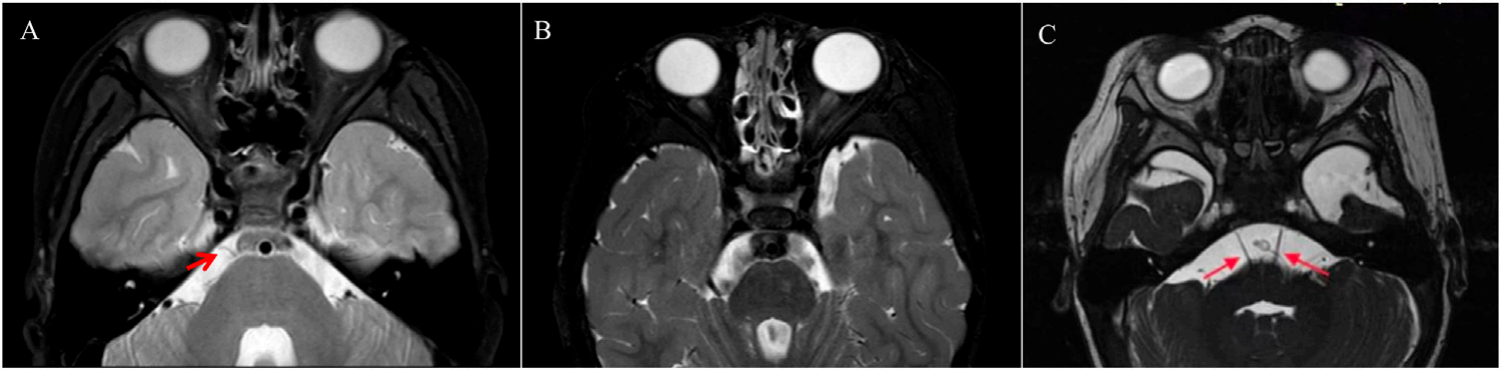
Axial MRI at the pontomedullary junction shows a normal right abducens nerve [**(A)** arrow]. Both abducens nerves could not be identified in a patient with bilateral DRS **(B)**. Both abducens nerves observed in a patient with DRS2 **(C)**.

**FIGURE 4:**
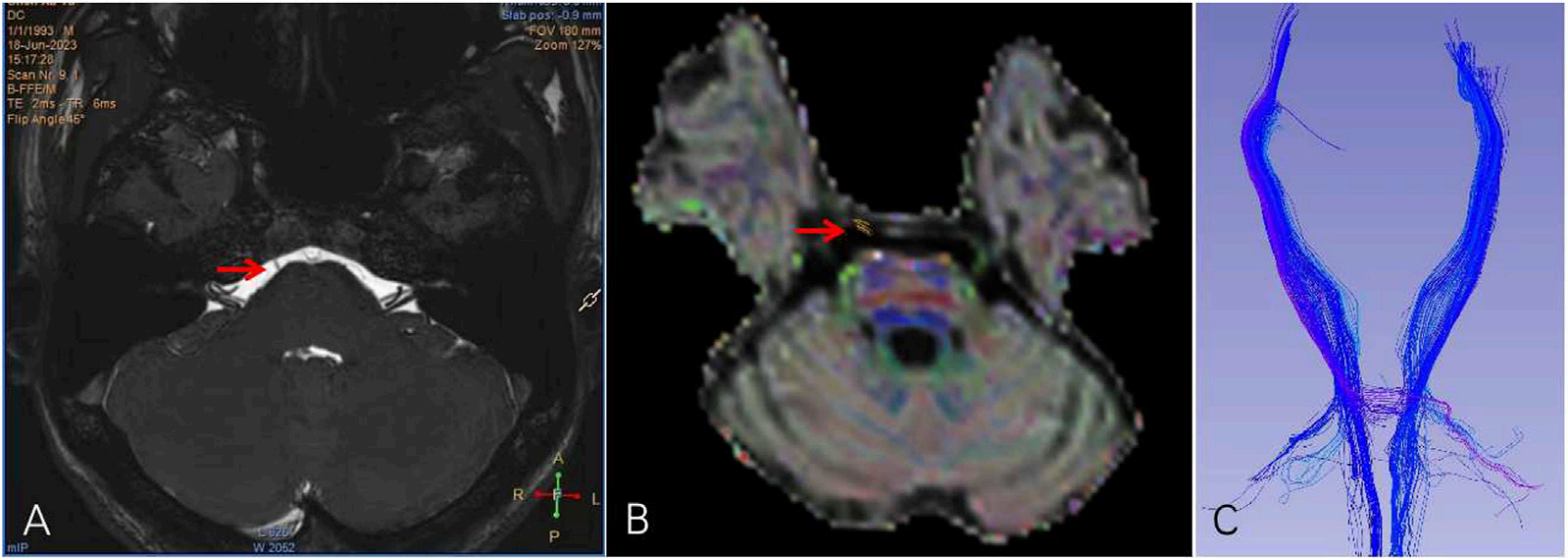
MRI and DTI images of a patient with DRS3. He had normal right abducens nerve [**(A, B)** arrow]. The left abducens nerve was absent, and the left corticospinal tract did not cross over to the opposite side **(C)**.

The abducens nerve was absent in 12 of the 14 patients with DRS1, including the patients with the *CHN1* and *SALL4* gene mutations, and 9 of the 17 with DRS3 in MRI images. Hypoplasia of the abducens nerve was observed in four patients with DRS2 (Figure 3). In addition, the projective fibers from the abducens neurons to the contralateral ocular motor neurons via the medial longitudinal fasciculus were also absent in those patients without abducens nerve in the DTI images (Figure 4).

### 3.3 Surgical outcomes

**FIGURE 5:**
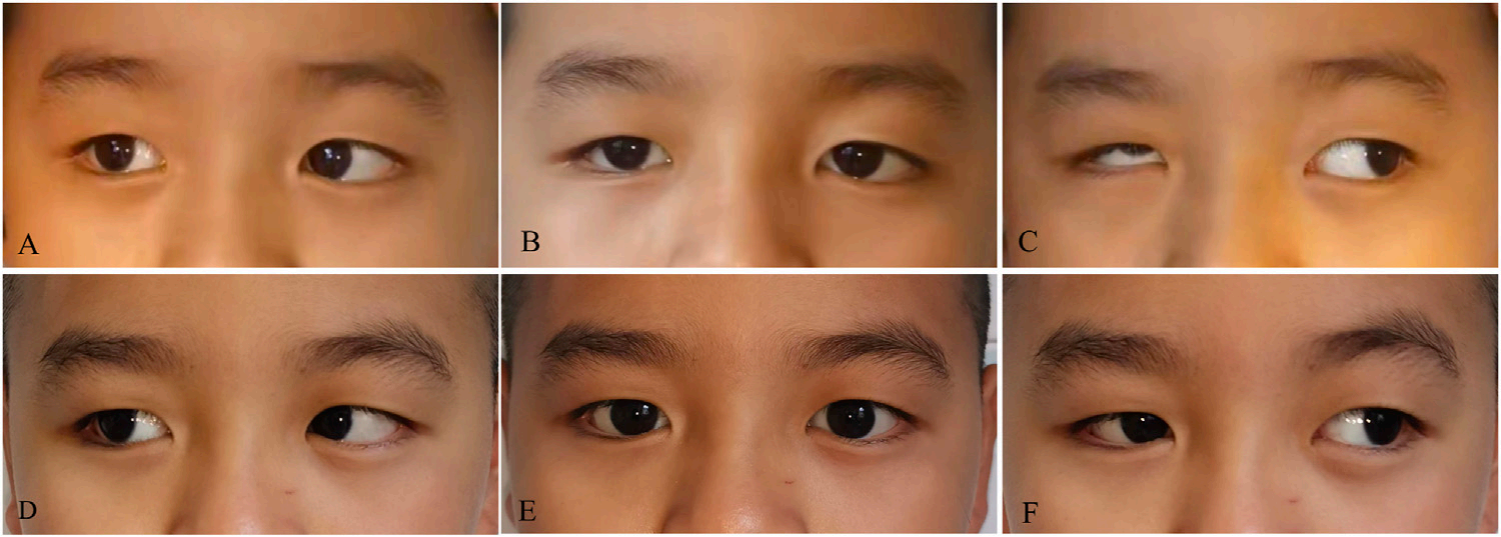
Pre- and post-operative images of a patient with DRS. There was restriction of abduction **(A)** in the right eye with exotropia **(B)**, narrowing and retraction of the globe, and upshoot on adduction **(C)**, which were improved after surgery **(D–F)**.

Thirty-five patients with DRS had their strabismus surgically corrected. The mean age at the time of surgery was 8.13 ± 3.61 years (range, 3–16 years). The mean postoperative follow-up period was 7.12 months (range, 6–19 months). The mean postoperative follow-up period was 7.12 months (range, 6–19 months). Seventeen patients with DRS1 underwent MR recession in conjunction with SRT. The esodeviation angle at the primary eye position was reduced from 33.21^△^ ± 5.86^△^ preoperatively to 3.33^△^ ± 2.39^△^ postoperatively (*P* < 0.01). Abduction of the affected eye has been improved, with an average of more than 2 mm beyond the midline. Four patients with DRS2 underwent a large amount of unilateral lateral rectus recession, with an average surgical dosage of 10 mm. The exodeviation was decreased from 50.67^△^ ± 6.56^△^ preoperatively to 2.96^△^ ± 2.77^△^ postoperatively (*P* < 0.01). However, the affected eye had limitations in both abduction and adduction, with a horizontal range of motion of less than 3 mm. Fifteen patients with DRS3 underwent unilateral MR recession combined with Y-splitting plus recession of LR for reducing shoots. Six patients with DRS3 underwent simultaneous recession of both MR and LR for globe retraction and shoots. After surgical operations, all patients with DRS3 had their strabismus, abnormal face turn, and up- and downshoot effectively improved (Figure 5). However, the improvement in eye movement amplitude after surgery was limited.

## 4 Discussion

DRS is thought to be caused by the underdevelopment of the sixth cranial nerve and its neurons during the embryonic stage. Genetic mutations may be involved. Three genes, *CHN1*, *MAFB,* and *SALL4* have been associated with DRS. These genes are involved in controlling the differentiation and patterning of cranial motor neurons and in controlling axon growth and guidance. *SALL4* is a transcription factor gene that is highly expressed in the developing midbrain, branchial arch, limbs, skeletal and ocular structures (Lim et al., 2008). Mutation in *SALL4* may cause syndromic DRS which is not only involved in cranial nerve and extraocular muscles, but also in radial maldevelopment (Duane radial-ray syndrome, DRRS) (Kohlhase et al., 2002; Borozdin et al., 2007). Individuals with *MAFB* variants may present with a syndromic phenotype, including inner ear agenesis and neurodevelopmental delay (Pascolini et al., 2022). *CHN1* is predominantly expressed in neurons and plays an important role in neuronal signal transduction mechanisms (Angelini et al., 2021). CHN1 encodes GTPase-activating proteins for the ras-related p21-rac and a phorbol ester receptor. The encoded protein has two isoforms sharing a RacGAP domain that interacts with and downregulates Rac activity, and a C1 domain that binds to diacylglycerol (DAG), and is predominantly expressed in neurons and plays an important role in neuronal signal transduction mechanisms (Miyake et al., 2008).

To date, 35 variants of *SALL4* and 15 variants of *CHN1* have been identified in patients with DRS. *CHN1* was identified from the DURS2 locus on chromosome 2 by linkage mapping on those families with DRS. In our patients, a *de novo* pathogenic variant of c.1432-2A>T in *SALL4* is detected in a patient with DRS1, and two novel pathogenic variants of c.377T>C (p. Ile126Thr) and c.659A>G (p. Glu220Gly) in *CHN1* are found from two pedigrees with DRS1. Most patients (37 in 42) are not found to have pathogenic variants in the DRS-related genes. Identified variants account for only 11% of patients, and the molecular etiologies of the vast majority of our patients remain unknown. A variant at codon 126 of Isoleucine has previously been reported to be pathogenic due to the replacement of the third base of T by G, which results in a wild-type amino acid of Isoleucine at codon 126 being replaced by a codon of Methionine (Miyake et al., 2008). The variant of c.377T > C (p. Ile126Thr) occurs in codon 126 as well. The difference of this variation with the previous reported variation is that Isoleucine is replaced by Threonine (Thr) due to the second base of T replaced by C at codon 126. Glu220 is highly conserved in different species and is located in the C1 domain. The previously reported variants of A223V, G228S and P252Q are also located in the C1 domain and are also highly conserved. Mutations occurring in the C1 domain are predicted to affect the binding of the C1 domain to the DAG, resulting in deregulation of normal oculomotor axon development.

In addition to the molecular etiology, we have evaluated the anatomic etiology of DRS using MRI and found that the abductor nerve is absent in most patients with DRS1 and DRS3 and hypoplasia is present in patients with DRS2, which may explain why patients with DRS suffer from abduction deficiency. The horizontal conjugate movement is also impaired due to absence of the projective fibers from the sixth nerve neuron to the contralateral the third nerve neuron via the medial longitudinal fasciculus.

Previous studies have shown that DRS1 is the most common type of DRS and may account for approximately 62% of patients with DRS (Masoomian et al., 2022), however, the most common type in our cohort was DRS3, followed by DRS1 and DRS2. This discrepancy may be related to source differences and sample size in our patients. With exceptions of a few patients with DRS3 who did not have strabismus at the primary eye position, a majority of patients (35 in 42 patients) underwent strabismus surgery for correcting ocular misalignment, abnormal facial turns and shoots. However, the limitations of abduction and adduction are not fully resolved, especially in DRS2 patients with large exodeviation according to our experience. The amplitude of the horizontal motion is only finitely improved after surgery due to the inert and fibrotic MR and LR muscles. Surgical procedures using SRT are effective in improving abduction, but should be used with caution in patients with severe globe retraction.

## 5 Conclusion

We reviewed the clinical characteristics, etiologies, and surgical outcomes of our patients with DRS retrospectively and identified three novel pathogenic variants in the *SALL4* and *CHN1* genes, respectively. These mutations will broaden the mutational spectrum of these two genes and contribute to understand the molecular etiology of DRS. We suggest that the cause of DRS may be explained by a spectrum of mechanical, neurological, and genetic abnormalities that exist independently or influence each other, resulting in a complex set of ocular or systemic anomalies that illustrate the variability and complexity of causes in DRS. In addition, the surgical treatment for DRS is still challenging and should be individualized.

## Data Availability

The original contributions presented in the study are publicly available. The data presented in the study are deposited in the NODE repository, accession number OEP005598.
